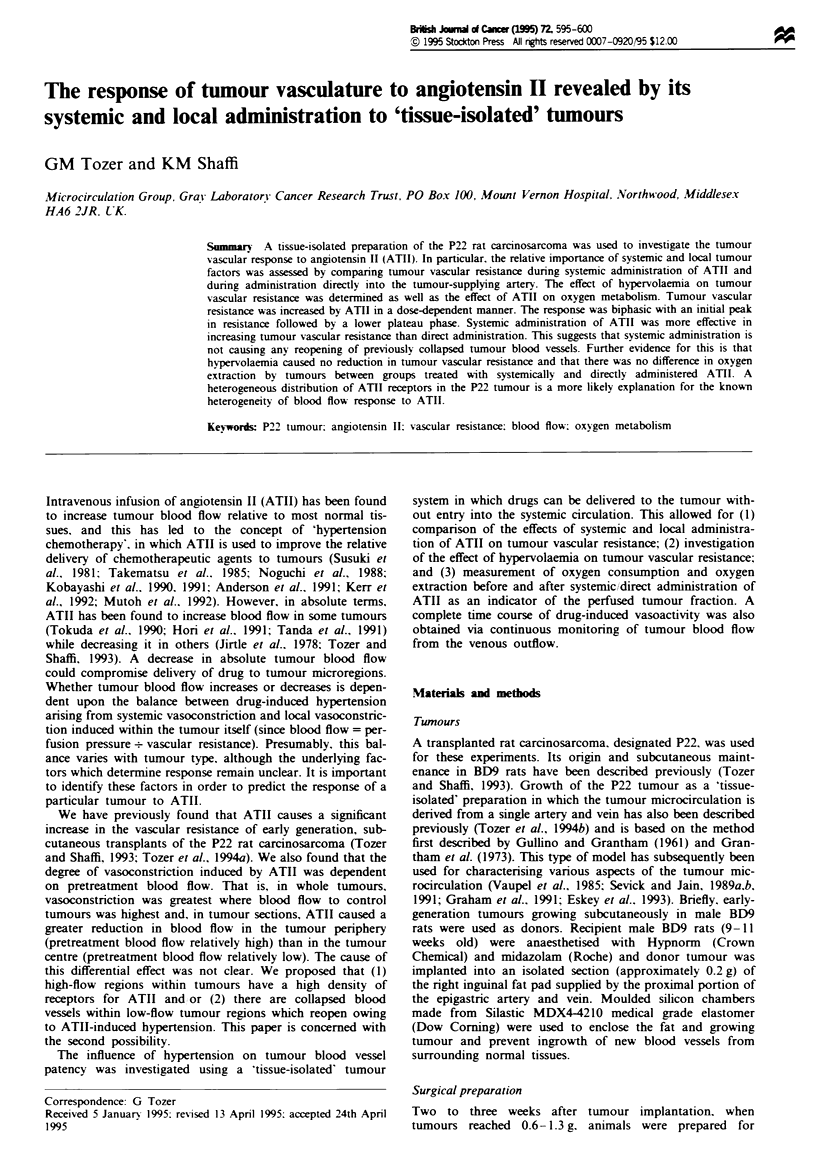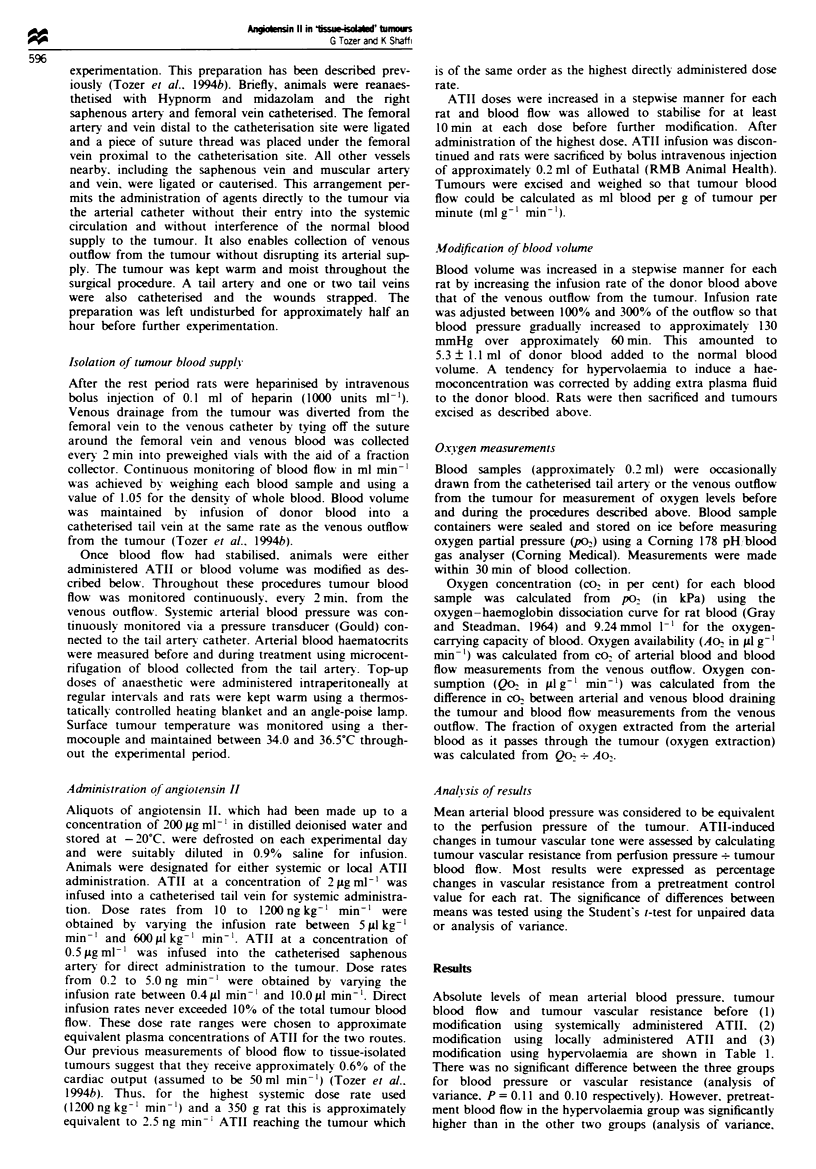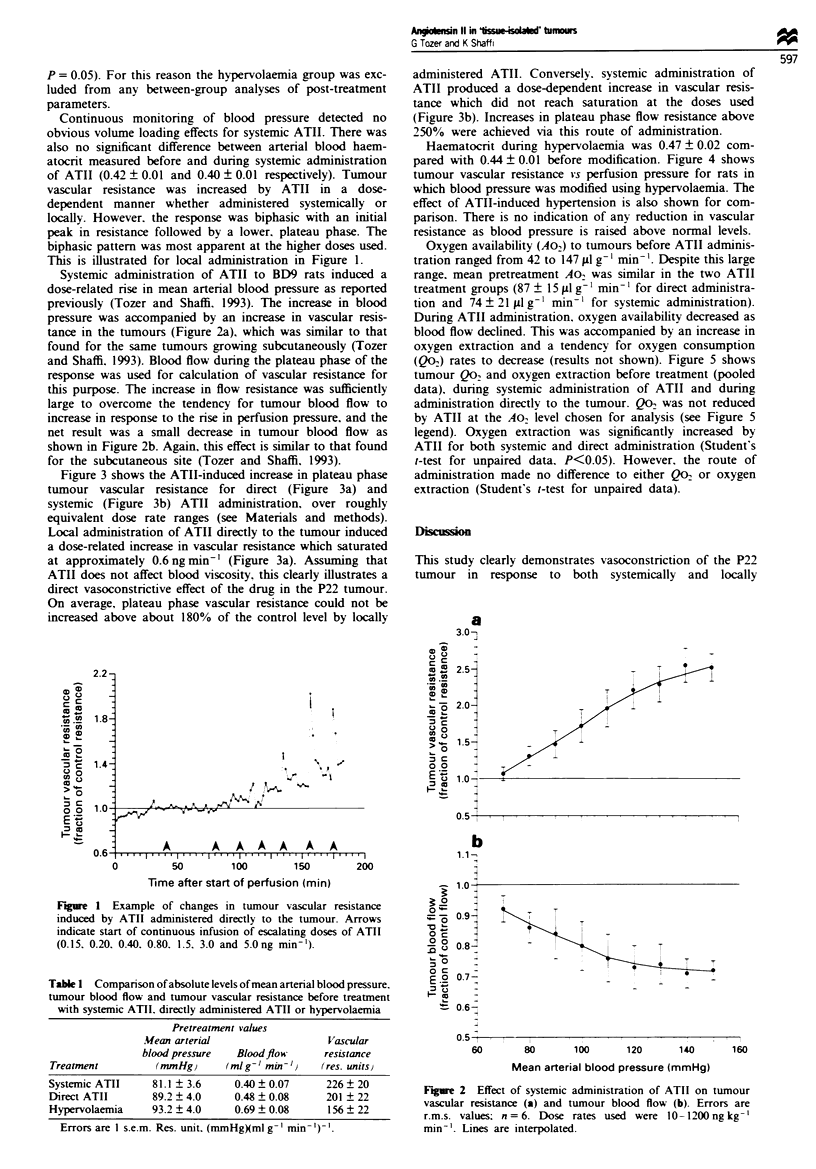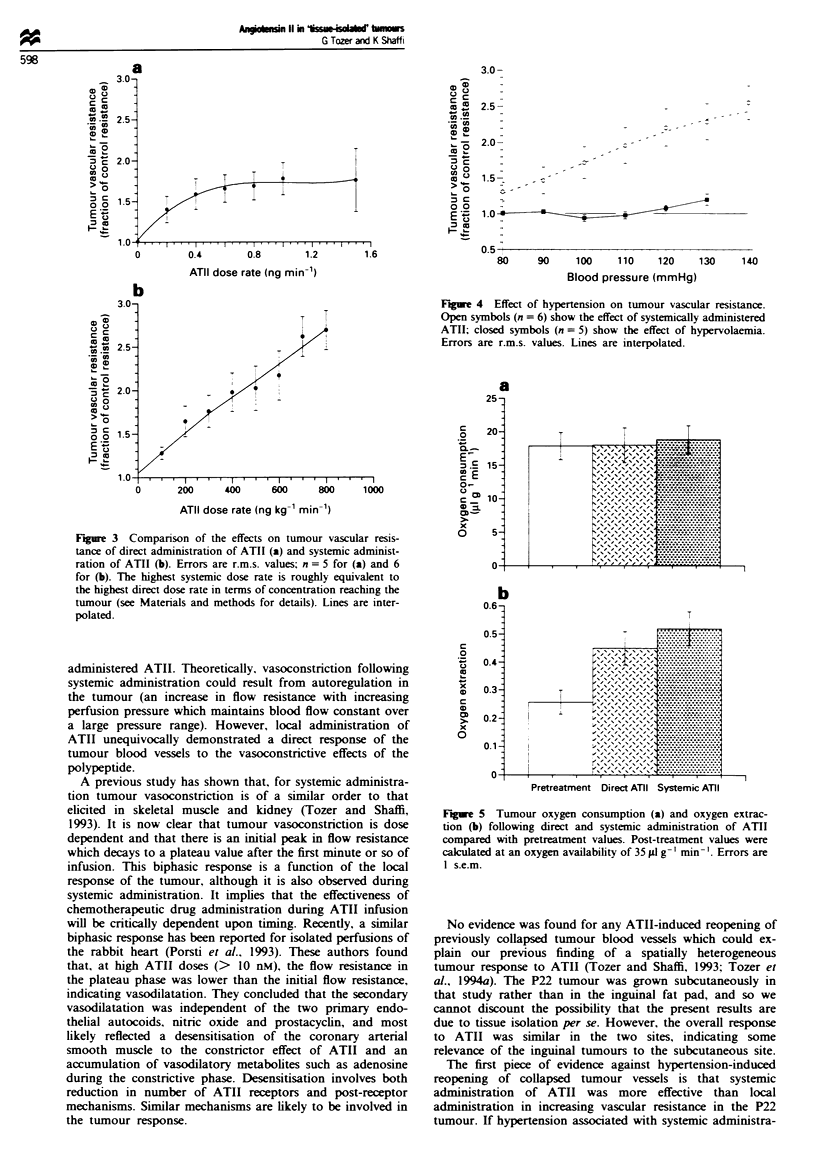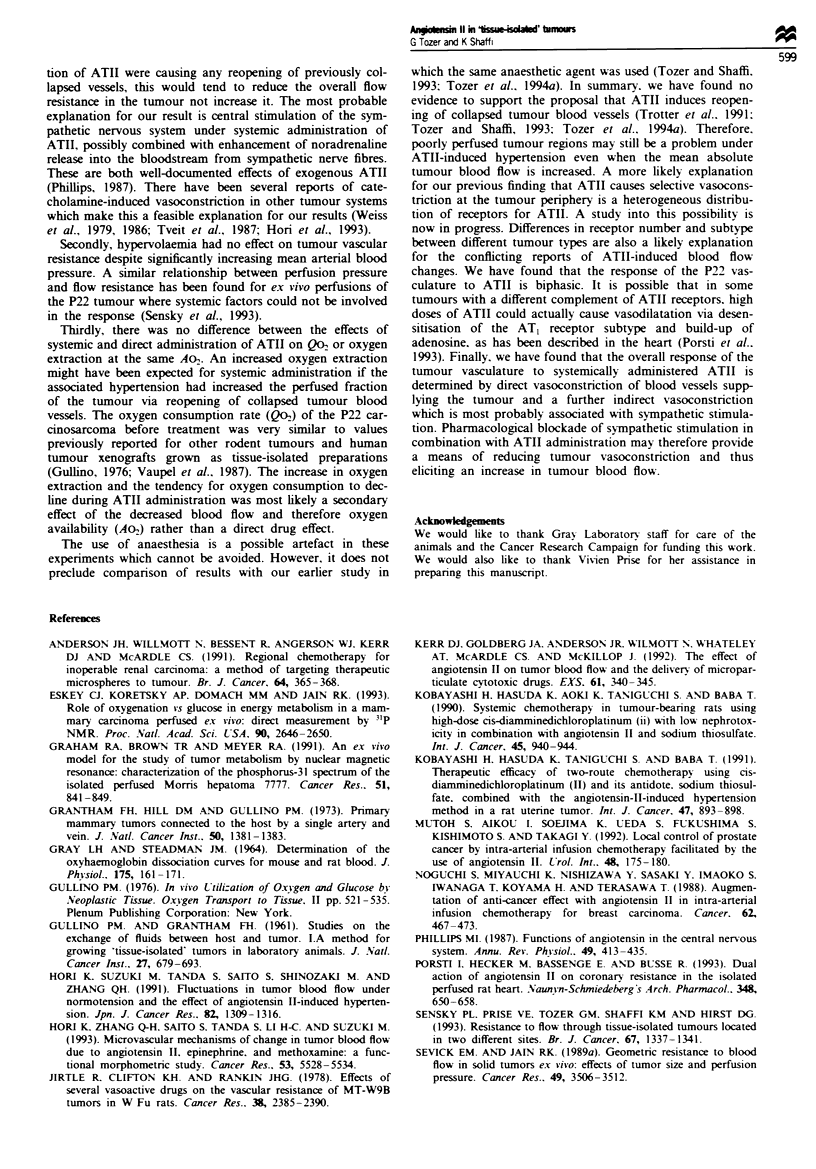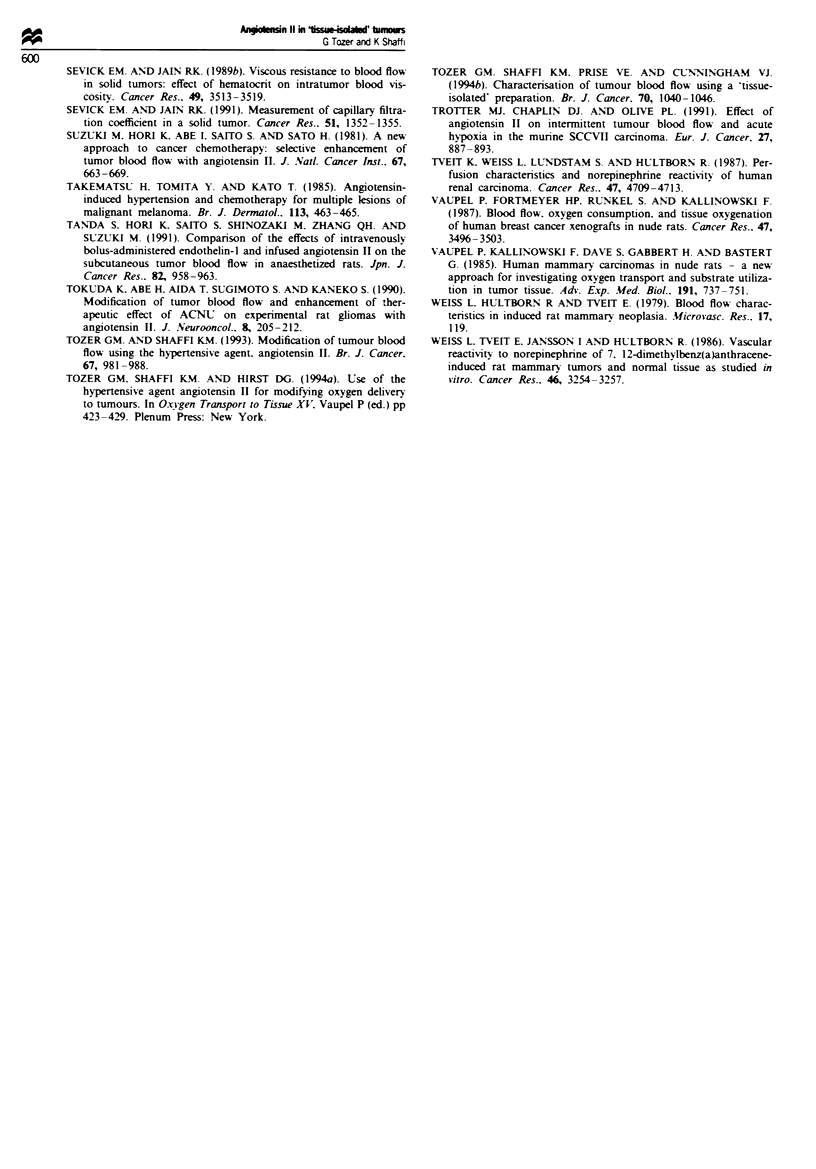# The response of tumour vasculature to angiotensin II revealed by its systemic and local administration to 'tissue-isolated' tumours.

**DOI:** 10.1038/bjc.1995.379

**Published:** 1995-09

**Authors:** G. M. Tozer, K. M. Shaffi

**Affiliations:** Gray Laboratory Cancer Research Trust, Mount Vernon Hospital, Northwood, Middlesex, UK.

## Abstract

A tissue-isolated preparation of the P22 rat carcinosarcoma was used to investigate the tumour vascular response to angiotensin II (ATII). In particular, the relative importance of systemic and local tumour factors was assessed by comparing tumour vascular resistance during systemic administration of ATII and during administration directly into the tumour-supplying artery. The effect of hypervolaemia on tumour vascular resistance was determined as well as the effect of ATII on oxygen metabolism. Tumour vascular resistance was increased by ATII in a dose-dependent manner. The response was biphasic with an initial peak in resistance followed by a lower plateau phase. Systemic administration of ATII was more effective in increasing tumour vascular resistance than direct administration. This suggests that systemic administration is not causing any reopening of previously collapsed tumour blood vessels. Further evidence for this is that hypervolaemia caused no reduction in tumour vascular resistance and that there was no difference in oxygen extraction by tumours between groups treated with systemically and directly administered ATII. A heterogeneous distribution of ATII receptors in the P22 tumour is a more likely explanation for the known heterogeneity of blood flow response to ATII.


© 1995 Stockton Press All rights reserved 0007-0920/95 $12.00

### The response of tumour vasculature to angiotensin II revealed by its systemic and local administration to 'tissue-isolated' tumours

GM Tozer and KM Shaffi

*Microcirculation Group, Gray Laboratory Cancer Research Trust, PO Box 100, Mount Vernon Hospital, Northwood, Middlesex HA6 2JR, UK.*

**Summary** A tissue-isolated preparation of the P22 rat carcinosarcoma was used to investigate the tumour vascular response to angiotensin II (ATII). In particular, the relative importance of systemic and local tumour factors was assessed by comparing tumour vascular resistance during systemic administration of ATII and during administration directly into the tumour-supplying artery. The effect of hypervolaemia on tumour vascular resistance was determined as well as the effect of ATII on oxygen metabolism. Tumour vascular resistance was increased by ATII in a dose-dependent manner. The response was biphasic with an initial peak in resistance followed by a lower plateau phase. Systemic administration of ATII was more effective in increasing tumour vascular resistance than direct administration. This suggests that systemic administration is not causing any reopening of previously collapsed tumour blood vessels. Further evidence for this is that hypervolaemia caused no reduction in tumour vascular resistance and that there was no difference in oxygen extraction by tumours between groups treated with systemically and directly administered ATII. A heterogeneous distribution of ATII receptors in the P22 tumour is a more likely explanation for the known heterogeneity of blood flow response to ATII.

**Keywords:** P22 tumour; angiotensin II; vascular resistance; blood flow; oxygen metabolism

Intravenous infusion of angiotensin II (ATII) has been found to increase tumour blood flow relative to most normal tissues, and this has led to the concept of 'hypertension chemotherapy', in which ATII is used to improve the relative delivery of chemotherapeutic agents to tumours (Susuki *et al.*, 1981; Takematsu *et al.*, 1985; Noguchi *et al.*, 1988; Kobayashi *et al.*, 1990, 1991; Anderson *et al.*, 1991; Kerr *et al.*, 1992; Mutoh *et al.*, 1992). However, in absolute terms, ATII has been found to increase blood flow in some tumours (Tokuda *et al.*, 1990; Hori *et al.*, 1991; Tanda *et al.*, 1991) while decreasing it in others (Jirtle *et al.*, 1978; Tozer and Shaffi, 1993). A decrease in absolute tumour blood flow could compromise delivery of drug to tumour microregions. Whether tumour blood flow increases or decreases is dependent upon the balance between drug-induced hypertension arising from systemic vasoconstriction and local vasoconstriction induced within the tumour itself (since blood flow = perfusion pressure ÷ vascular resistance). Presumably, this balance varies with tumour type, although the underlying factors which determine response remain unclear. It is important to identify these factors in order to predict the response of a particular tumour to ATII.

We have previously found that ATII causes a significant increase in the vascular resistance of early generation, subcutaneous transplants of the P22 rat carcinosarcoma (Tozer and Shaffi, 1993; Tozer *et al.*, 1994*a*). We also found that the degree of vasoconstriction induced by ATII was dependent on pretreatment blood flow. That is, in whole tumours, vasoconstriction was greatest where blood flow to control tumours was highest and, in tumour sections, ATII caused a greater reduction in blood flow in the tumour periphery (pretreatment blood flow relatively high) than in the tumour centre (pretreatment blood flow relatively low). The cause of this differential effect was not clear. We proposed that (1) high-flow regions within tumours have a high density of receptors for ATII and/or (2) there are collapsed blood vessels within low-flow tumour regions which reopen owing to ATII-induced hypertension. This paper is concerned with the second possibility.

The influence of hypertension on tumour blood vessel patency was investigated using a 'tissue-isolated' tumour system in which drugs can be delivered to the tumour without entry into the systemic circulation. This allowed for (1) comparison of the effects of systemic and local administration of ATII on tumour vascular resistance; (2) investigation of the effect of hypervolaemia on tumour vascular resistance; and (3) measurement of oxygen consumption and oxygen extraction before and after systemic/direct administration of ATII as an indicator of the perfused tumour fraction. A complete time course of drug-induced vasoactivity was also obtained via continuous monitoring of tumour blood flow from the venous outflow.

### Materials and methods

### Tumours

A transplanted rat carcinosarcoma, designated P22, was used for these experiments. Its origin and subcutaneous maintenance in BD9 rats have been described previously (Tozer and Shaffi, 1993). Growth of the P22 tumour as a 'tissue-isolated' preparation in which the tumour microcirculation is derived from a single artery and vein has also been described previously (Tozer *et al.*, 1994*b*) and is based on the method first described by Gullino and Grantham (1961) and Grantham *et al.* (1973). This type of model has subsequently been used for characterising various aspects of the tumour microcirculation (Vaupel *et al.*, 1985; Sevick and Jain, 1989*a*,*b*, 1991; Graham *et al.*, 1991; Eskey *et al.*, 1993). Briefly, early-generation tumours growing subcutaneously in male BD9 rats were used as donors. Recipient male BD9 rats (9-11 weeks old) were anaesthetised with Hypnorm (Crown Chemical) and midazolam (Roche) and donor tumour was implanted into an isolated section (approximately 0.2 g) of the right inguinal fat pad supplied by the proximal portion of the epigastric artery and vein. Moulded silicon chambers made from Silastic MDX4-4210 medical grade elastomer (Dow Corning) were used to enclose the fat and growing tumour and prevent ingrowth of new blood vessels from surrounding normal tissues.

### Surgical preparation

Two to three weeks after tumour implantation, when tumours reached 0.6-1.3 g, animals were prepared for

Correspondence: G Tozer

Received 5 January 1995; revised 13 April 1995; accepted 24th April 1995

experimentation. This preparation has been described previously (Tozer *et al.*, 1994*b*). Briefly, animals were reanaesthetised with Hypnorm and midazolam and the right saphenous artery and femoral vein catheterised. The femoral artery and vein distal to the catheterisation site were ligated and a piece of suture thread was placed under the femoral vein proximal to the catheterisation site. All other vessels nearby, including the saphenous vein and muscular artery and vein, were ligated or cauterised. This arrangement permits the administration of agents directly to the tumour via the arterial catheter without their entry into the systemic circulation and without interference of the normal blood supply to the tumour. It also enables collection of venous outflow from the tumour without disrupting its arterial supply. The tumour was kept warm and moist throughout the surgical procedure. A tail artery and one or two tail veins were also catheterised and the wounds strapped. The preparation was left undisturbed for approximately half an hour before further experimentation.

### *Isolation of tumour blood supply*

After the rest period rats were heparinised by intravenous bolus injection of 0.1 ml of heparin (1000 units $ml^{-1}$). Venous drainage from the tumour was diverted from the femoral vein to the venous catheter by tying off the suture around the femoral vein and venous blood was collected every 2 min into preweighed vials with the aid of a fraction collector. Continuous monitoring of blood flow in ml $min^{-1}$ was achieved by weighing each blood sample and using a value of 1.05 for the density of whole blood. Blood volume was maintained by infusion of donor blood into a catheterised tail vein at the same rate as the venous outflow from the tumour (Tozer *et al.*, 1994*b*).

Once blood flow had stabilised, animals were either administered ATII or blood volume was modified as described below. Throughout these procedures tumour blood flow was monitored continuously, every 2 min, from the venous outflow. Systemic arterial blood pressure was continuously monitored via a pressure transducer (Gould) connected to the tail artery catheter. Arterial blood haematocrits were measured before and during treatment using microcentrifugation of blood collected from the tail artery. Top-up doses of anaesthetic were administered intraperitoneally at regular intervals and rats were kept warm using a thermostatically controlled heating blanket and an angle-poise lamp. Surface tumour temperature was monitored using a thermocouple and maintained between 34.0 and 36.5°C throughout the experimental period.

### *Administration of angiotensin II*

Aliquots of angiotensin II, which had been made up to a concentration of 200 $\mu g\ ml^{-1}$ in distilled deionised water and stored at −20°C, were defrosted on each experimental day and were suitably diluted in 0.9% saline for infusion. Animals were designated for either systemic or local ATII administration. ATII at a concentration of 2 $\mu g\ ml^{-1}$ was infused into a catheterised tail vein for systemic administration. Dose rates from 10 to 1200 $ng\ kg^{-1}\ min^{-1}$ were obtained by varying the infusion rate between 5 $\mu l\ kg^{-1}\ min^{-1}$ and 600 $\mu l\ kg^{-1}\ min^{-1}$. ATII at a concentration of 0.5 $\mu g\ ml^{-1}$ was infused into the catheterised saphenous artery for direct administration to the tumour. Dose rates from 0.2 to 5.0 ng $min^{-1}$ were obtained by varying the infusion rate between 0.4 $\mu l\ min^{-1}$ and 10.0 $\mu l\ min^{-1}$. Direct infusion rates never exceeded 10% of the total tumour blood flow. These dose rate ranges were chosen to approximate equivalent plasma concentrations of ATII for the two routes. Our previous measurements of blood flow to tissue-isolated tumours suggest that they receive approximately 0.6% of the cardiac output (assumed to be 50 ml $min^{-1}$) (Tozer *et al.*, 1994*b*). Thus, for the highest systemic dose rate used (1200 $ng\ kg^{-1}\ min^{-1}$) and a 350 g rat this is approximately equivalent to 2.5 ng $min^{-1}$ ATII reaching the tumour which is of the same order as the highest directly administered dose rate.

ATII doses were increased in a stepwise manner for each rat and blood flow was allowed to stabilise for at least 10 min at each dose before further modification. After administration of the highest dose, ATII infusion was discontinued and rats were sacrificed by bolus intravenous injection of approximately 0.2 ml of Euthatal (RMB Animal Health). Tumours were excised and weighed so that tumour blood flow could be calculated as ml blood per g of tumour per minute ($ml\ g^{-1}\ min^{-1}$).

### *Modification of blood volume*

Blood volume was increased in a stepwise manner for each rat by increasing the infusion rate of the donor blood above that of the venous outflow from the tumour. Infusion rate was adjusted between 100% and 300% of the outflow so that blood pressure gradually increased to approximately 130 mmHg over approximately 60 min. This amounted to $5.3 \pm 1.1$ ml of donor blood added to the normal blood volume. A tendency for hypervolaemia to induce a haemoconcentration was corrected by adding extra plasma fluid to the donor blood. Rats were then sacrificed and tumours excised as described above.

### *Oxygen measurements*

Blood samples (approximately 0.2 ml) were occasionally drawn from the catheterised tail artery or the venous outflow from the tumour for measurement of oxygen levels before and during the procedures described above. Blood sample containers were sealed and stored on ice before measuring oxygen partial pressure ($po_2$) using a Corning 178 pH/blood gas analyser (Corning Medical). Measurements were made within 30 min of blood collection.

Oxygen concentration ($co_2$ in per cent) for each blood sample was calculated from $po_2$ (in kPa) using the oxygen–haemoglobin dissociation curve for rat blood (Gray and Steadman, 1964) and 9.24 mmol $l^{-1}$ for the oxygen-carrying capacity of blood. Oxygen availability ($Ao_2$ in $\mu l\ g^{-1}\ min^{-1}$) was calculated from $co_2$ of arterial blood and blood flow measurements from the venous outflow. Oxygen consumption ($Qo_2$ in $\mu l\ g^{-1}\ min^{-1}$) was calculated from the difference in $co_2$ between arterial and venous blood draining the tumour and blood flow measurements from the venous outflow. The fraction of oxygen extracted from the arterial blood as it passes through the tumour (oxygen extraction) was calculated from $Qo_2 \div Ao_2$.

### *Analysis of results*

Mean arterial blood pressure was considered to be equivalent to the perfusion pressure of the tumour. ATII-induced changes in tumour vascular tone were assessed by calculating tumour vascular resistance from perfusion pressure ÷ tumour blood flow. Most results were expressed as percentage changes in vascular resistance from a pretreatment control value for each rat. The significance of differences between means was tested using the Student's *t*-test for unpaired data or analysis of variance.

### Results

Absolute levels of mean arterial blood pressure, tumour blood flow and tumour vascular resistance before (1) modification using systemically administered ATII, (2) modification using locally administered ATII and (3) modification using hypervolaemia are shown in Table 1. There was no significant difference between the three groups for blood pressure or vascular resistance (analysis of variance, $P = 0.11$ and 0.10 respectively). However, pretreatment blood flow in the hypervolaemia group was significantly higher than in the other two groups (analysis of variance,

$P = 0.05$). For this reason the hypervolaemia group was excluded from any between-group analyses of post-treatment parameters.

Continuous monitoring of blood pressure detected no obvious volume loading effects for systemic ATII. There was also no significant difference between arterial blood haematocrit measured before and during systemic administration of ATII ($0.42 \pm 0.01$ and $0.40 \pm 0.01$ respectively). Tumour vascular resistance was increased by ATII in a dose-dependent manner whether administered systemically or locally. However, the response was biphasic with an initial peak in resistance followed by a lower, plateau phase. The biphasic pattern was most apparent at the higher doses used. This is illustrated for local administration in Figure 1.

Systemic administration of ATII to BD9 rats induced a dose-related rise in mean arterial blood pressure as reported previously (Tozer and Shaffi, 1993). The increase in blood pressure was accompanied by an increase in vascular resistance in the tumours (Figure 2a), which was similar to that found for the same tumours growing subcutaneously (Tozer and Shaffi, 1993). Blood flow during the plateau phase of the response was used for calculation of vascular resistance for this purpose. The increase in flow resistance was sufficiently large to overcome the tendency for tumour blood flow to increase in response to the rise in perfusion pressure, and the net result was a small decrease in tumour blood flow as shown in Figure 2b. Again, this effect is similar to that found for the subcutaneous site (Tozer and Shaffi, 1993).

Figure 3 shows the ATII-induced increase in plateau phase tumour vascular resistance for direct (Figure 3a) and systemic (Figure 3b) ATII administration, over roughly equivalent dose rate ranges (see Materials and methods). Local administration of ATII directly to the tumour induced a dose-related increase in vascular resistance which saturated at approximately $0.6\ \mathrm{ng\ min^{-1}}$ (Figure 3a). Assuming that ATII does not affect blood viscosity, this clearly illustrates a direct vasoconstrictive effect of the drug in the P22 tumour. On average, plateau phase vascular resistance could not be increased above about 180% of the control level by locally administered ATII. Conversely, systemic administration of ATII produced a dose-dependent increase in vascular resistance which did not reach saturation at the doses used (Figure 3b). Increases in plateau phase flow resistance above 250% were achieved via this route of administration.

Haematocrit during hypervolaemia was $0.47 \pm 0.02$ compared with $0.44 \pm 0.01$ before modification. Figure 4 shows tumour vascular resistance *vs* perfusion pressure for rats in which blood pressure was modified using hypervolaemia. The effect of ATII-induced hypertension is also shown for comparison. There is no indication of any reduction in vascular resistance as blood pressure is raised above normal levels.

Oxygen availability ($AO_2$) to tumours before ATII administration ranged from 42 to $147\ \mu\mathrm{l\ g^{-1}\ min^{-1}}$. Despite this large range, mean pretreatment $AO_2$ was similar in the two ATII treatment groups ($87 \pm 15\ \mu\mathrm{l\ g^{-1}\ min^{-1}}$ for direct administration and $74 \pm 21\ \mu\mathrm{l\ g^{-1}\ min^{-1}}$ for systemic administration). During ATII administration, oxygen availability decreased as blood flow declined. This was accompanied by an increase in oxygen extraction and a tendency for oxygen consumption ($QO_2$) rates to decrease (results not shown). Figure 5 shows tumour $QO_2$ and oxygen extraction before treatment (pooled data), during systemic administration of ATII and during administration directly to the tumour. $QO_2$ was not reduced by ATII at the $AO_2$ level chosen for analysis (see Figure 5 legend). Oxygen extraction was significantly increased by ATII for both systemic and direct administration (Student's $t$-test for unpaired data, $P<0.05$). However, the route of administration made no difference to either $QO_2$ or oxygen extraction (Student's $t$-test for unpaired data).

### Discussion

This study clearly demonstrates vasoconstriction of the P22 tumour in response to both systemically and locally

**Figure 1** Example of changes in tumour vascular resistance induced by ATII administered directly to the tumour. Arrows indicate start of continuous infusion of escalating doses of ATII (0.15, 0.20, 0.40, 0.80, 1.5, 3.0 and $5.0\ \mathrm{ng\ min^{-1}}$).

**Table 1** Comparison of absolute levels of mean arterial blood pressure, tumour blood flow and tumour vascular resistance before treatment with systemic ATII, directly administered ATII or hypervolaemia

| | *Pretreatment values* | | |
|---|---|---|---|
| *Treatment* | *Mean arterial blood pressure (mmHg)* | *Blood flow ($ml\ g^{-1}\ min^{-1}$)* | *Vascular resistance (res. units)* |
| Systemic ATII | $81.1 \pm 3.6$ | $0.40 \pm 0.07$ | $226 \pm 20$ |
| Direct ATII | $89.2 \pm 4.0$ | $0.48 \pm 0.08$ | $201 \pm 22$ |
| Hypervolaemia | $93.2 \pm 4.0$ | $0.69 \pm 0.08$ | $156 \pm 22$ |

Errors are 1 s.e.m. Res. unit, $(\mathrm{mmHg})(\mathrm{ml\ g^{-1}\ min^{-1}})^{-1}$.

**Figure 2** Effect of systemic administration of ATII on tumour vascular resistance (**a**) and tumour blood flow (**b**). Errors are r.m.s. values; $n = 6$. Dose rates used were $10-1200\ \mathrm{ng\ kg^{-1}\ min^{-1}}$. Lines are interpolated.

**Figure 3** Comparison of the effects on tumour vascular resistance of direct administration of ATII (**a**) and systemic administration of ATII (**b**). Errors are r.m.s. values; $n = 5$ for (**a**) and 6 for (**b**). The highest systemic dose rate is roughly equivalent to the highest direct dose rate in terms of concentration reaching the tumour (see Materials and methods for details). Lines are interpolated.

**Figure 4** Effect of hypertension on tumour vascular resistance. Open symbols ($n = 6$) show the effect of systemically administered ATII; closed symbols ($n = 5$) show the effect of hypervolaemia. Errors are r.m.s. values. Lines are interpolated.

**Figure 5** Tumour oxygen consumption (**a**) and oxygen extraction (**b**) following direct and systemic administration of ATII compared with pretreatment values. Post-treatment values were calculated at an oxygen availability of 35 µl g$^{-1}$ min$^{-1}$. Errors are 1 s.e.m.

administered ATII. Theoretically, vasoconstriction following systemic administration could result from autoregulation in the tumour (an increase in flow resistance with increasing perfusion pressure which maintains blood flow constant over a large pressure range). However, local administration of ATII unequivocally demonstrated a direct response of the tumour blood vessels to the vasoconstrictive effects of the polypeptide.

A previous study has shown that, for systemic administration tumour vasoconstriction is of a similar order to that elicited in skeletal muscle and kidney (Tozer and Shaffi, 1993). It is now clear that tumour vasoconstriction is dose dependent and that there is an initial peak in flow resistance which decays to a plateau value after the first minute or so of infusion. This biphasic response is a function of the local response of the tumour, although it is also observed during systemic administration. It implies that the effectiveness of chemotherapeutic drug administration during ATII infusion will be critically dependent upon timing. Recently, a similar biphasic response has been reported for isolated perfusions of the rabbit heart (Porsti *et al*., 1993). These authors found that, at high ATII doses (> 10 nM), the flow resistance in the plateau phase was lower than the initial flow resistance, indicating vasodilatation. They concluded that the secondary vasodilatation was independent of the two primary endothelial autocoids, nitric oxide and prostacyclin, and most likely reflected a desensitisation of the coronary arterial smooth muscle to the constrictor effect of ATII and an accumulation of vasodilatory metabolites such as adenosine during the constrictive phase. Desensitisation involves both reduction in number of ATII receptors and post-receptor mechanisms. Similar mechanisms are likely to be involved in the tumour response.

No evidence was found for any ATII-induced reopening of previously collapsed tumour blood vessels which could explain our previous finding of a spatially heterogeneous tumour response to ATII (Tozer and Shaffi, 1993; Tozer *et al*., 1994*a*). The P22 tumour was grown subcutaneously in that study rather than in the inguinal fat pad, and so we cannot discount the possibility that the present results are due to tissue isolation *per se*. However, the overall response to ATII was similar in the two sites, indicating some relevance of the inguinal tumours to the subcutaneous site.

The first piece of evidence against hypertension-induced reopening of collapsed tumour vessels is that systemic administration of ATII was more effective than local administration in increasing vascular resistance in the P22 tumour. If hypertension associated with systemic administration of ATII were causing any reopening of previously collapsed vessels, this would tend to reduce the overall flow resistance in the tumour not increase it. The most probable explanation for our result is central stimulation of the sympathetic nervous system under systemic administration of ATII, possibly combined with enhancement of noradrenaline release into the bloodstream from sympathetic nerve fibres. These are both well-documented effects of exogenous ATII (Phillips, 1987). There have been several reports of catecholamine-induced vasoconstriction in other tumour systems which make this a feasible explanation for our results (Weiss *et al.*, 1979, 1986; Tveit *et al.*, 1987; Hori *et al.*, 1993).

Secondly, hypervolaemia had no effect on tumour vascular resistance despite significantly increasing mean arterial blood pressure. A similar relationship between perfusion pressure and flow resistance has been found for *ex vivo* perfusions of the P22 tumour where systemic factors could not be involved in the response (Sensky *et al.*, 1993).

Thirdly, there was no difference between the effects of systemic and direct administration of ATII on $Q_{O_2}$ or oxygen extraction at the same $A_{O_2}$. An increased oxygen extraction might have been expected for systemic administration if the associated hypertension had increased the perfused fraction of the tumour via reopening of collapsed tumour blood vessels. The oxygen consumption rate ($Q_{O_2}$) of the P22 carcinosarcoma before treatment was very similar to values previously reported for other rodent tumours and human tumour xenografts grown as tissue-isolated preparations (Gullino, 1976; Vaupel *et al.*, 1987). The increase in oxygen extraction and the tendency for oxygen consumption to decline during ATII administration was most likely a secondary effect of the decreased blood flow and therefore oxygen availability ($A_{O_2}$) rather than a direct drug effect.

The use of anaesthesia is a possible artefact in these experiments which cannot be avoided. However, it does not preclude comparison of results with our earlier study in which the same anaesthetic agent was used (Tozer and Shaffi, 1993; Tozer *et al.*, 1994*a*). In summary, we have found no evidence to support the proposal that ATII induces reopening of collapsed tumour blood vessels (Trotter *et al.*, 1991; Tozer and Shaffi, 1993; Tozer *et al.*, 1994*a*). Therefore, poorly perfused tumour regions may still be a problem under ATII-induced hypertension even when the mean absolute tumour blood flow is increased. A more likely explanation for our previous finding that ATII causes selective vasoconstriction at the tumour periphery is a heterogeneous distribution of receptors for ATII. A study into this possibility is now in progress. Differences in receptor number and subtype between different tumour types are also a likely explanation for the conflicting reports of ATII-induced blood flow changes. We have found that the response of the P22 vasculature to ATII is biphasic. It is possible that in some tumours with a different complement of ATII receptors, high doses of ATII could actually cause vasodilatation via desensitisation of the $AT_1$ receptor subtype and build-up of adenosine, as has been described in the heart (Porsti *et al.*, 1993). Finally, we have found that the overall response of the tumour vasculature to systemically administered ATII is determined by direct vasoconstriction of blood vessels supplying the tumour and a further indirect vasoconstriction which is most probably associated with sympathetic stimulation. Pharmacological blockade of sympathetic stimulation in combination with ATII administration may therefore provide a means of reducing tumour vasoconstriction and thus eliciting an increase in tumour blood flow.

**Acknowledgements**
We would like to thank Gray Laboratory staff for care of the animals and the Cancer Research Campaign for funding this work. We would also like to thank Vivien Prise for her assistance in preparing this manuscript.

**References**

ANDERSON JH, WILLMOTT N, BESSENT R, ANGERSON WJ, KERR DJ AND McARDLE CS. (1991). Regional chemotherapy for inoperable renal carcinoma: a method of targeting therapeutic microspheres to tumour. *Br. J. Cancer*, **64**, 365-368.

ESKEY CJ, KORETSKY AP, DOMACH MM AND JAIN RK. (1993). Role of oxygenation *vs* glucose in energy metabolism in a mammary carcinoma perfused *ex vivo*: direct measurement by $^{31}$P NMR. *Proc. Natl. Acad. Sci. USA*, **90**, 2646-2650.

GRAHAM RA, BROWN TR AND MEYER RA. (1991). An *ex vivo* model for the study of tumor metabolism by nuclear magnetic resonance: characterization of the phosphorus-31 spectrum of the isolated perfused Morris hepatoma 7777. *Cancer Res.*, **51**, 841-849.

GRANTHAM FH, HILL DM AND GULLINO PM. (1973). Primary mammary tumors connected to the host by a single artery and vein. *J. Natl. Cancer Inst.*, **50**, 1381-1383.

GRAY LH AND STEADMAN JM. (1964). Determination of the oxyhaemoglobin dissociation curves for mouse and rat blood. *J. Physiol.*, **175**, 161-171.

GULLINO PM. (1976). *In vivo Utilization of Oxygen and Glucose by Neoplastic Tissue. Oxygen Transport to Tissue*, II pp. 521-535. Plenum Publishing Corporation: New York.

GULLINO PM. AND GRANTHAM FH. (1961). Studies on the exchange of fluids between host and tumor. I.A method for growing 'tissue-isolated' tumors in laboratory animals. *J. Natl. Cancer Inst.*, **27**, 679-693.

HORI K, SUZUKI M, TANDA S, SAITO S, SHINOZAKI M AND ZHANG QH. (1991). Fluctuations in tumor blood flow under normotension and the effect of angiotensin II-induced hypertension. *Jpn. J. Cancer Res.*, **82**, 1309-1316.

HORI K, ZHANG Q-H, SAITO S, TANDA S, LI H-C. AND SUZUKI M. (1993). Microvascular mechanisms of change in tumor blood flow due to angiotensin II, epinephrine, and methoxamine: a functional morphometric study. *Cancer Res.*, **53**, 5528-5534.

JIRTLE R, CLIFTON KH. AND RANKIN JHG. (1978). Effects of several vasoactive drugs on the vascular resistance of MT-W9B tumors in W Fu rats. *Cancer Res.*, **38**, 2385-2390.

KERR DJ, GOLDBERG JA, ANDERSON JR, WILMOTT N, WHATELEY AT, McARDLE CS. AND McKILLOP J. (1992). The effect of angiotensin II on tumor blood flow and the delivery of microparticulate cytotoxic drugs. *EXS*, **61**, 340-345.

KOBAYASHI H, HASUDA K, AOKI K, TANIGUCHI S. AND BABA T. (1990). Systemic chemotherapy in tumour-bearing rats using high-dose cis-diamminedichloroplatinum (ii) with low nephrotoxicity in combination with angiotensin II and sodium thiosulfate. *Int. J. Cancer*, **45**, 940-944.

KOBAYASHI H, HASUDA K, TANIGUCHI S. AND BABA T. (1991). Therapeutic efficacy of two-route chemotherapy using cis-diamminedichloroplatinum (II) and its antidote, sodium thiosulfate, combined with the angiotensin-II-induced hypertension method in a rat uterine tumor. *Int. J. Cancer*, **47**, 893-898.

MUTOH S, AIKOU I, SOEJIMA K, UEDA S, FUKUSHIMA S, KISHIMOTO S. AND TAKAGI Y. (1992). Local control of prostate cancer by intra-arterial infusion chemotherapy facilitated by the use of angiotensin II. *Urol. Int.*, **48**, 175-180.

NOGUCHI S, MIYAUCHI K, NISHIZAWA Y, SASAKI Y, IMAOKO S, IWANAGA T, KOYAMA H. AND TERASAWA T. (1988). Augmentation of anti-cancer effect with angiotensin II in intra-arterial infusion chemotherapy for breast carcinoma. *Cancer*, **62**, 467-473.

PHILLIPS MI. (1987). Functions of angiotensin in the central nervous system. *Annu. Rev. Physiol.*, **49**, 413-435.

PORSTI I, HECKER M, BASSENGE E. AND BUSSE R. (1993). Dual action of angiotensin II on coronary resistance in the isolated perfused rat heart. *Naunyn-Schmiedeberg's Arch. Pharmacol.*, **348**, 650-658.

SENSKY PL, PRISE VE, TOZER GM, SHAFFI KM AND HIRST DG. (1993). Resistance to flow through tissue-isolated tumours located in two different sites. *Br. J. Cancer*, **67**, 1337-1341.

SEVICK EM. AND JAIN RK. (1989*a*). Geometric resistance to blood flow in solid tumors *ex vivo*: effects of tumor size and perfusion pressure. *Cancer Res.*, **49**, 3506-3512.

SEVICK EM. AND JAIN RK. (1989*b*). Viscous resistance to blood flow in solid tumors: effect of hematocrit on intratumor blood viscosity. *Cancer Res.*, **49**, 3513–3519.

SEVICK EM. AND JAIN RK. (1991). Measurement of capillary filtration coefficient in a solid tumor. *Cancer Res.*, **51**, 1352–1355.

SUZUKI M. HORI K. ABE I. SAITO S. AND SATO H. (1981). A new approach to cancer chemotherapy: selective enhancement of tumor blood flow with angiotensin II. *J. Natl. Cancer Inst.*, **67**, 663–669.

TAKEMATSU H. TOMITA Y. AND KATO T. (1985). Angiotensin-induced hypertension and chemotherapy for multiple lesions of malignant melanoma. *Br. J. Dermatol.*, **113**, 463–465.

TANDA S. HORI K. SAITO S. SHINOZAKI M. ZHANG QH. AND SUZUKI M. (1991). Comparison of the effects of intravenously bolus-administered endothelin-1 and infused angiotensin II on the subcutaneous tumor blood flow in anaesthetized rats. *Jpn. J. Cancer Res.*, **82**, 958–963.

TOKUDA K. ABE H. AIDA T. SUGIMOTO S. AND KANEKO S. (1990). Modification of tumor blood flow and enhancement of therapeutic effect of ACNU on experimental rat gliomas with angiotensin II. *J. Neurooncol.*, **8**, 205–212.

TOZER GM. AND SHAFFI KM. (1993). Modification of tumour blood flow using the hypertensive agent, angiotensin II. *Br. J. Cancer*, **67**, 981–988.

TOZER GM. SHAFFI KM. AND HIRST DG. (1994*a*). Use of the hypertensive agent angiotensin II for modifying oxygen delivery to tumours. In *Oxygen Transport to Tissue XV*, Vaupel P (ed.) pp 423–429. Plenum Press: New York.

TOZER GM. SHAFFI KM. PRISE VE. AND CUNNINGHAM VJ. (1994*b*). Characterisation of tumour blood flow using a 'tissue-isolated' preparation. *Br. J. Cancer*, **70**, 1040–1046.

TROTTER MJ. CHAPLIN DJ. AND OLIVE PL. (1991). Effect of angiotensin II on intermittent tumour blood flow and acute hypoxia in the murine SCCVII carcinoma. *Eur. J. Cancer*, **27**, 887–893.

TVEIT K. WEISS L. LUNDSTAM S. AND HULTBORN R. (1987). Perfusion characteristics and norepinephrine reactivity of human renal carcinoma. *Cancer Res.*, **47**, 4709–4713.

VAUPEL P. FORTMEYER HP. RUNKEL S. AND KALLINOWSKI F. (1987). Blood flow, oxygen consumption, and tissue oxygenation of human breast cancer xenografts in nude rats. *Cancer Res.*, **47**, 3496–3503.

VAUPEL P. KALLINOWSKI F. DAVE S. GABBERT H. AND BASTERT G. (1985). Human mammary carcinomas in nude rats – a new approach for investigating oxygen transport and substrate utilization in tumor tissue. *Adv. Exp. Med. Biol.*, **191**, 737–751.

WEISS L. HULTBORN R AND TVEIT E. (1979). Blood flow characteristics in induced rat mammary neoplasia. *Microvasc. Res.*, **17**, 119.

WEISS L. TVEIT E. JANSSON I AND HULTBORN R. (1986). Vascular reactivity to norepinephrine of 7, 12-dimethylbenz(a)anthracene-induced rat mammary tumors and normal tissue as studied *in vitro*. *Cancer Res.*, **46**, 3254–3257.